# Modelled lung deposition and retention of welding fume particles in occupational scenarios: a comparison to doses used in vitro

**DOI:** 10.1007/s00204-022-03247-9

**Published:** 2022-02-21

**Authors:** Sarah McCarrick, Hanna L. Karlsson, Ulrika Carlander

**Affiliations:** Institute of Environmental Medicine, Karolinska Institutet, 171 77 Stockholm, Sweden

**Keywords:** Welding, Exposure, Particle deposition, Lung dose, In vitro

## Abstract

Translating particle dose from in vitro systems to relevant human exposure remains a major challenge for the use of in vitro studies in assessing occupational hazard and risk of particle exposure. This study aimed to model the lung deposition and retention of welding fume particles following occupational scenarios and subsequently compare the lung doses to those used in vitro. We reviewed published welding fume concentrations and size distributions to identify input values simulating real-life exposure scenarios in the multiple path particle dosimetry (MPPD) model. The majority of the particles were reported to be below 0.1 μm and mass concentrations ranged between 0.05 and 45 mg/m^3^. Following 6-h exposure to 5 mg/m^3^ with a count median diameter of 50 nm, the tracheobronchial lung dose (0.89 µg/cm^2^) was found to exceed the in vitro cytotoxic cell dose (0.125 µg/cm^2^) previously assessed by us in human bronchial epithelial cells (HBEC-3kt). However, the tracheobronchial retention decreased rapidly when no exposure occurred, in contrast to the alveolar retention which builds-up over time and exceeded the in vitro cytotoxic cell dose after 1.5 working week. After 1 year, the tracheobronchial and alveolar retention was estimated to be 1.15 and 2.85 µg/cm^2^, respectively. Exposure to low-end aerosol concentrations resulted in alveolar retention comparable to cytotoxic in vitro dose in HBEC-3kt after 15–20 years of welding. This study demonstrates the potential of combining real-life exposure data with particle deposition modelling to improve the understanding of in vitro concentrations in the context of human occupational exposure.

**Supplementary Information:**

The online version contains supplementary material available at 10.1007/s00204-022-03247-9.

## Introduction

Welding fumes are created as a by-product of the welding process and contain toxic metals including chromium, nickel and manganese that are of concern for occupational health. Approximately 10 million people are estimated to somehow be exposed to welding fumes in their occupational setting (IARC 2017), where the metal-containing fumes have been linked to several health outcomes including bronchitis, respiratory irritation, and inflammation (Antonini 2003; Zeidler-Erdely et al. 2012; Riccelli et al. 2020). Welding fumes are further established to be carcinogenic to humans by the International Agency for Research on Cancer (IARC 2017). Yet, no common welding specific occupational exposure limit (OEL) has been established within the European union (EU), instead 8-h OEL for (inorganic) dust or separate OELs for specific metals such as manganese and hexavalent chromium are often used (Sjögren et al. 2021). Recently, an evaluation of OEL for welding fume in the EU has been initiated by the European chemicals agency.

Using in vitro systems, we and others have demonstrated toxic effects in cultured cells exposed to welding fume particles, including cytotoxicity, oxidative stress, DNA damage and inflammatory effects (Antonini et al. 1999, 2005; McNeilly et al. 2004; Leonard et al. 2010; Shoeb et al. 2017; McCarrick et al. 2019, 2021; Hedberg et al. 2021). In vitro toxicity test methods offer the potential for efficient, economical and ethical hazard testing as an alternative to animal testing. However, extrapolating human health risk from chemical exposure based on in vitro data is a challenging task. One important aspect lies in the in vitro dose selection, where scientific justification and consideration for real world exposure is crucial to provide useful data for toxicological hazard and risk assessment (Oberdörster and Kuhlbusch 2018; Romeo et al. 2020).

Aerosol exposure concentration is frequently considered as an acceptable measure for lung dose in experimental studies, risk assessments and in the derivation of regulatory limit values. Nevertheless, this may be deceiving since the exposure has been argued to be a poor predictor of delivered dose in the lung (Oller and Oberdörster 2016). Lung dose is dependent on several factors including aerosol concentration, respiratory parameters and particle characteristics affecting the mobility and behavior of the particles (Oberdörster et al. 2005; Hofmann 2020). As argueed by Schmid and Cassee (2017), the concept of delivered dose is of principal significance for any type of particle exposure and the relevant measure for toxicological dose–response analysis for human risk assessment. Therefore, data on the deposition and clearance of particles in the human respiratory system are key parameters to assess the association between human exposure and in vitro dose levels.

As emphasized in the review by Riccelli et al. (2020), the quantification of welding particle deposition and its clearance are essential for evaluating their health risks. In contrast to readily measured exposure concentrations, delivered dose is difficult to quantify. Efforts have been made to determine the fate of welding fume particles in vivo, including assessments of lung deposition, clearance and extrapulmonary distribution (Kalliomäki et al. 1978; Antonini et al. 2010, 2011; Falcone et al. 2017; Stanislawska et al. 2020). Nonetheless, experimental determination of particle deposition is limited to primarily total lung deposition with regional deposition of less accuracy. To refine health risk assessment, dosimetry models can serve as an alternative to provide more detailed information on local deposition patterns in the human lung. These models can also aid in the translational framework needed to relate nominal concentrations inducing biological response to a relevant human exposure level, i.e., in vitro to in vivo extrapolation (IVIVE) (Anjilvel and Asgharian 1995; Miller et al. 2016; Romeo et al. 2020). A widely accepted model providing detailed dosimetry data is the multiple-path particle dosimetry (MPPD) model. By adopting the MPPD model, the total, regional and lobar deposition fraction per airway can be attained and the influence of particle specific aerosol characteristics and respiratory parameters can be assessed (Anjilvel and Asgharian 1995; Miller et al. 2016). The estimated regional lung doses can further be used to correlate in vivo particle exposure levels to in vitro dose levels, as previously done by Gangwal et al. (2011) among others.

The aim of this study was to investigate the deposition of welding fume particles in the lung following real-life occupational exposure scenarios and to explore how this can be related to doses used in vitro. To find relevant exposure data, a comprehensive literature search of available studies was performed. We reviewed the articles and selected relevant articles based on the following criteria: (1) occupational exposure data for welding fumes measured within industrial workplaces or laboratories; and (2) size distribution of the particles with at least 4 size fractions. Secondly, the multiple-path particle dosimetry (MPPD) model was used to run hypothetical exposure scenarios and investigate the role of particle size distribution, particle density, exposure concentrations, body position and breathing patterns for deposition and retention in the tracheobronchial and alveolar region of the lung, respectively. Finally, the modelled doses were linked to doses and findings from our own in vitro studies. The quantification of particle deposition and clearance is essential for finding links between real-life exposure to welding fume and toxic effects studied in vitro, and thus has implications in the risk assessment of workers exposed to welding fumes.

## Method

### Literature search

The search was conducted during June 2021 in Web of Science. The search terms included the following: weld* AND fume* AND exposure*. The articles dating from 2010 and onwards were evaluated with regard to relevance for our study.

In a first screening, we included studies meeting the following inclusion criteria regarding exposure assessment: publications with original occupational quantitative measurements of solely welding fumes (not mixed work), measurement of total fume and thus not only single metal constituents and aerosol concentrations reported in number or mass. Only peer-reviewed publications written in English were included. We excluded reviews and studies based on pre-defined simulations, exposures based on biomarkers or questionaries and other non-relevant topics (such as in vivo and in vitro studies) on the basis of abstract and/or full text. To be able to generate results useful for the MPPD modelling, publications reporting welding concentrations in other metrics than mass or number (e.g., surface area) were not considered further.

After preliminary screening, studies considered relevant were assessed for eligibility for MPPD modelling regarding reported size distribution with the following inclusion criteria: original quantitative size distribution measurements with at least 4 size fractions. Studies with no size distribution data or size distributions with less than 4 size fractions (e.g., inhalable and respirable fraction) or based on TEM-imaging were further excluded.

### Data extraction and reporting

From all the articles that met the inclusion criteria, information of interest was extracted and summarized. General information was collected on population investigated, location, welding method and base material. Data on exposure concentrations and size distributions were gathered from the publications without processing to the extent as possible, unless otherwise specified. If certain information/data were not available in the publication, these were stated as not reported (NR), unless specifically stated.

The method used to determine size distribution including metric and size range of the instrument were noted. To ensure consistency in data extraction, the following rules were specified: if geometric mean aerodynamic diameter (GMAD) or count median diameter (CMD) with GSD were reported in the studies these were extracted without processing. If only a graph of the size distribution curve was available, a GMD was calculated by collecting datapoints from the graph using WebPlotDigitizer (https://automeris.io/WebPlotDigitizer/). If size was reported in mass frequency distribution in the different size fractions, this was reported without processing. If size distribution was stated as mass/number in different size fractions, these were converted to % of total mass/number based on total particulate matter (PM) reported in the study, or if not available the sum of all fractions reported. All values calculated by us are marked with *.

Data on aerosol concentration measurements including the instrument used for quantification, the sampling matrix, location of measurement and duration of measurement was collected. Concentration in mass (mg/m^3^) or number (particles/cm^3^) were extracted in the format stated in the publication including mean (geometric or arithmetic), median, range or single measurement with associated variance. If two concentration values were given, they were both included whereas more than two means or medians were given as a range.

### MPPD modelling

The MPPD dosimetry model v3.04 (ARA) was used. MPPD is a mechanistic model that calculates deposition and clearance based on user-provided input on airway morphometry, particle properties, exposure scenario and clearance rates (Anjilvel and Asgharian 1995; Miller et al. 2016). To determine the deposition of welding fume particles in the lung, we initially selected a baseline set of MPPD inputs based on a combination of data on physiological parameters from the ICRP report (ICRP 1994) and welding particle specific data based on the results of our literature compilation. The MPPD model offers two sets of exposure conditions: constant or variable. Our simulations were performed using constant exposure conditions with or without clearance. We further varied selected parameters (size, exposure concentration, particle density, workload, body position) systematically to evaluate the key determinants for deposition of welding fume particles in the lung.

The results were visualized using GraphPad Prism 8.0.1 (GraphPad Inc., La Jolla, CA).

### Baseline input values

The airway morphometry selected in the MPPD model was the Yeh–Schum 5-lobe lung model based on data by Yeh and Schum (1980) with variable path lengths among the five lobes but symmetric branching within each lobe to better reproduce the asymmetry of the airway branching pattern.

The particles were assumed to be spherical (aspect ratio of 1). The baseline density was set to 7.9 g/cm^3^, corresponding to the density of iron (Fe). Baseline size distribution was based on the results of the literature compilation. Function for adjustment of particle inhalability was used to correct for particles ability to reach and enter the upper respiratory tract (Asgharian et al. 2018).

The baseline breathing pattern was selected to simulate light exercise, referred to as medium workload, corresponding to oronasal-mouth breather with 20 breaths per minute and a tidal volume of 1250 mL. Body orientation was set to upright. The baseline exposure concentrations was set to the general OEL used in the regulation of welding fumes corresponding to 5 mg/m^3^ according to IARC (2017). This is equivalent to the OEL for inorganic inhalable dust used for welding fumes in Sweden (Swedish Work Environment Authority 2018). Default clearance values were selected in the MPPD model.

### MPPD simulations

To investigate the influence of size distribution on deposition fractions, the MPPD model was run for deposition only (no clearance). A wide range of size distributions were selected from the results of the literature compilation to compare to the baseline (also selected from literature).

To study the welding fume particle retention (deposition and clearance), we simulated 1 week of exposure, assuming five working days with 6 h full exposure. This is in line with IARC, reporting the median duration of exposure to be 40 h/week for welders with 70% exposed full-time (IARC 2017). The MPPD calculations were performed, in addition to the baseline values, with a variation in concentration, size distribution, density (1/4 g/cm^3^), work burden (light and heavy corresponding to 12/26 breaths per minute and 625/1600 tidal volume, respectively) and body position (on back, leaning forward). A wide range of size distributions was selected based on the results of the literature review. Further, the lowest and the highest exposure concentration reported in the literature review were used to simulate the far ends of the exposure range in relation to the OEL. To be able to use the OEL as a baseline, we decided to only use the range of concentrations reported in mass. However, we recognize that other lung deposition metrics (such as particle number and particle surface area) are important for understanding the health risks (Oberdörster et al. 2005; Schmid and Stoeger 2016).

Finally, a 1-year simulation (52 weeks, 5 working days/week with 6 h/day of exposure) was run for the baseline input values as well as a 45-year simulation (2340 weeks, 5 working days/week with 6 h/day of exposure) for the low end-exposure level with remaining baseline values.

### Calculations of retention per surface area

The results on tracheobronchial and alveolar retention that were given by the MPPD model were further converted to retention per surface area by dividing the retention by total surface area of the different regions. The total tracheobronchial (3220 cm^2^) and alveolar surface area (786,000 cm^2^) was obtained from the MPPD model by summing the tracheobronchial surface area for generation 1–17 or the pulmonary surface area for generation 16–25, respectively. Together, the total area of the tracheobronchial and alveolar region corresponds to approximately 79 m^2^.

### Comparison of MPPD calculated lung doses to in vitro

The surface area lung retention reached following the different exposure scenarios was directly compared to in vitro doses previously used by us to assess toxicity of welding fumes generated from stainless steel flux cored arc welding in human bronchial epithelial cells (HBEC-3kt) under submerged conditions (McCarrick et al. 2021). The in vitro doses were given as μg/mL but were converted to μg/cm^2^ by assuming 100 μL exposure medium and a growth area of 0.32 cm^2^ in a 96-well plate. The nominal dose was based on full sedimentation and bioavailability, whilst the cellular dose assumed 5% uptake based on uptake measurements in McCarrick et al. (2021). The half maximal effective concentration (EC50) of the most cytotoxic welding fume tested, i.e., the dose eliciting a reduction of 50% cell viability, was included to represent an in vitro dose inducing a response. However, since this was the most toxic welding fume tested, it can be considered as a type of worst-case exposure.

## Results

### Literature search

**Fig. 1:**
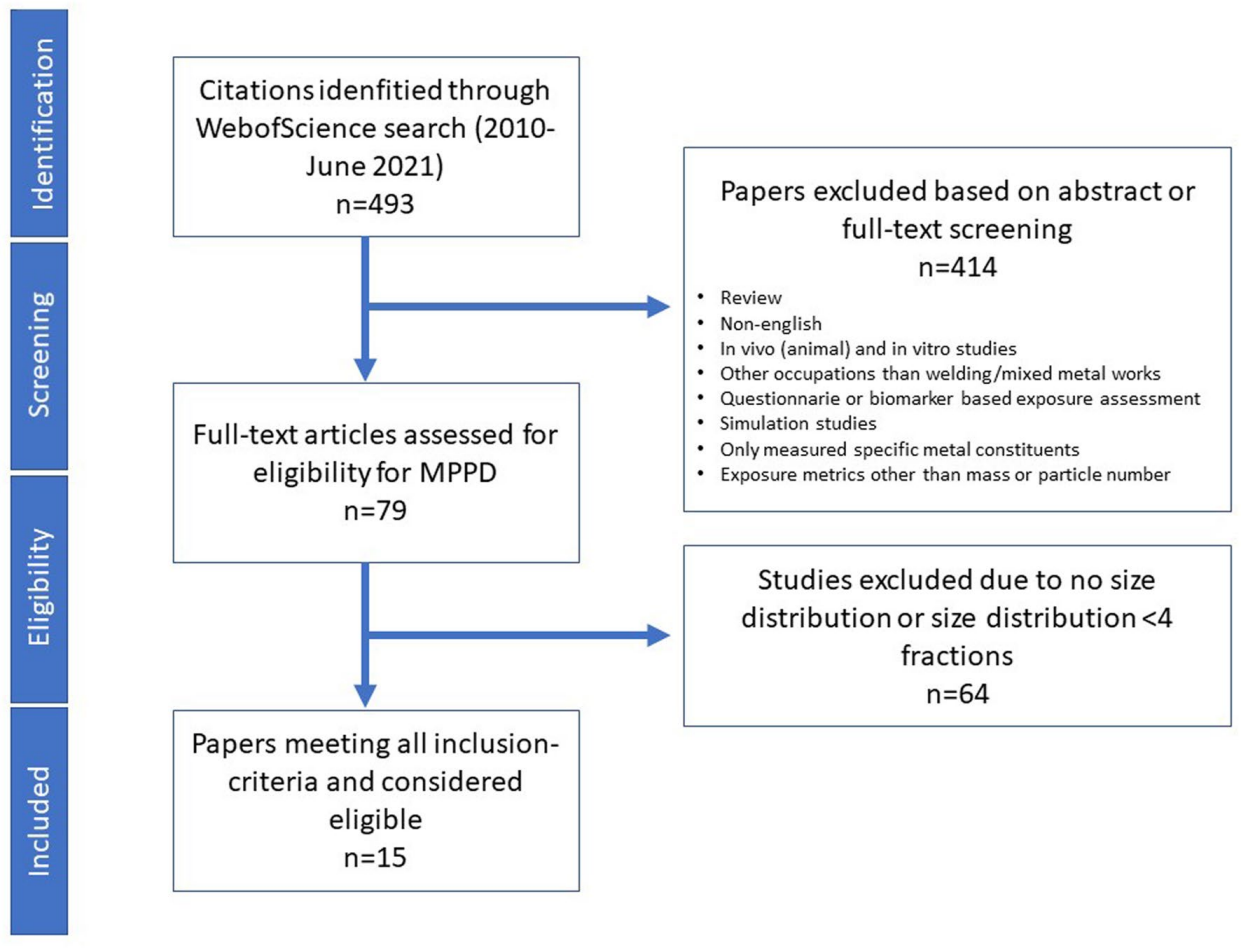
Literature search flow

The literature search generated 493 articles, out of which 79 were identified with a suitable exposure assessment of welding fume particles (414 excluded). Out of these, 15 articles met the established criteria of reporting size distribution with at least 4 size fractions to be used to generate input values for the MPPD modelling (64 excluded). The literature search flow is illustrated in Fig. 1.

The selected studies from the literature search provide data in a variety of welding occupations as well as methods and materials used in the welding process, see Table 1 or for more details Table S1 and S2. The studies identified generally fell into two broad categories: experimental investigations and field studies. The experimental studies involved somewhat controlled settings (worker, location, time frame of welding etc.) where the field studies were more uncontrolled and may therefore to a larger extent include the presence of confounders (such as co-exposures from surrounding operations), however this can be argued to provide a more real-world exposure. The studies differed in measurement approaches including both gravimetric and real-time measurements as well as both personal and static/stationary measurements. The measurement instruments used for measuring concentrations and size distribution in the various studies have different operation principles and measurement ranges. Measurements were further reported in breathing zone and a variety of other distances from the welding source. Based on these factors, the concentrations and size distributions reported in the studies may not be fully comparable. Even so, the results presented here may be considered representative for general levels and size distributions of welding fume particles in occupational settings.

**Table 1:** Summary welding methods and conditions and size distribution

References	Population; location, welding method, base material, ventilation, PPE	mg/m^3^ ± SD	Particles/cm^3^; GSD	GMD (µm); GSD	MMAD (µm); GSD	Comments/additional data
Cena et al. (2016)	Experimental; NR, GMAW, MS, no ventilation, NR	45 ± 2.2 (BZ) 9 ± 2.2 (2 m) AM	2.7 × 10^6^ (BZ) 2.6 × 10^6^ (2 m) NR	0.20*	0.35; 1.5 (BZ) 0.3; 1.367 (2 m)	Bimodal distribution with small second peak around 0.01 µm
Debia et al. (2014)	Apprentice welders; Canada, FCAW/GMAW/GTAW/SMAW, Aluminium/steel/SS, General and local exhaust ventilation, NR	–	0.065–0.17 × 10^6^; 1.2–1.7 GM	NR	NR	PM0.1: 48–88 num% (welding activity), 75–93 num% (whole welding period) Main modes around: 50/214/98/50 (welding activity), 30/30/98/30 (whole welding period) for FCAW/GMAW/GTAW/SMAW
Graczyk et al. (2016)	Apprentice welders; Switzerland, TIG, aluminium, pulsing ventilation, non-ventilated helmet	0.72 (BZ) 0.67 (0.6 m) Median	1.7 × 10^6^; 2.4(BZ) 1.1 × 10^6^;1.9 (0.6 m) 0.77 × 10^6^; 1.6 (0.6 m) GM	0.045;1.6 (BZ) 0.051; 1.5 (0.6 m) 0.069; 1.9 (0.6 m)	NR	< 0.041 µm: 50 num% PM0.1: 92 num%
Ham et al. (2012)	Welders; NR, GMAW, MS, general ventilation, NR	0.48/1.1 (total, gravimetric) 0.20/0.35 (PM1, RT) GM	0.31/3.1 × 10^6^; 2.7/3.1 (total, SMPS) 0.034/0.054 × 10^6^ (total, CPC) GM	NR	NR	3D-format graph: no data extracted PM0.1: 64–68 num%*
Hedmer et al. (2014)	Welders in manufacturing; Sweden, GMAW/MIG/MAG, MS, mixed ventilation, mixed PPE	1.3 GSD: 2.9 GM	–	NR	NR	PM0.25: 53 wt% of PM10 PM1: 73 wt%* of PM10 PM2.5:83 wt %* of PM10
Insley (2019)	Welders in metal products fabrication; Pennsylvania, US, FCAW, CS, general ventilation, NR	0.25 GSD: 3.4 (0.9–1.5 m) 0.14 GSD: 3.5 (2.1–2.7 m) GM	0.039 × 10^6^; 2.4 (0.9–1.5 m) 0.040 × 10^6^; 2.3 (2.1–2.7 m) GM	NR	NR	PM1: 80 wt%* PM2.5: 80 wt%* PM10: 92 wt%*
Kirichenko et al. (2019)	Experimental; NR, arc welding, low alloyed steel, no ventilation, NR Particles measured after 15 min settling period or other distances: data not extracted, see original article	–	0.003–0.0045 × 10^6^; NR (Total, height 0.8 m/15 min settling) NR	NR	NR	PM0.3: 78–83 num%* of PM10 PM1: 99.8–99.9 num%* of PM10 PM3: 99.9 num%* of PM10 at height 0.8 m from source and 15 min settling time
Lai et al. (2016)	Shipyard welders; Taiwan, TIG, Galvanized metal, NR, masks	0.90 (PM10, environmental) 0.049;0.032 (PM2.5, personal) Mean	0.22 × 10^6^ (< 160 nm) 0.00089 × 10^6^ (> 542 nm) NR	0.041*	NR	CMD: Bimodal 0.014–0.015 µm 0.13–0.14 µm PM0.1: 9 wt% of PM10* PM2.5: 84 wt% of PM10*
Lehnert et al. (2012)	Mixed welders; Germany, FCAW/GMAW /SMAW/TIG/miscellaneous, SS/MS/other, mixed ventilation, mixed PPE	2.5 (excl PARPS) Median	0.12 × 10^6^ median	FCAW: 0.10* GMAW: 0.090* SMAW: 0.067* TIG: 0.042*	NR	PM0.1 μm: 54 num%* Concentrations: FCAW > GMAW > SMAW > TIG
Lin et al. (2015)	Fitness equipment manufacturers; Taiwan, NR (manual and automatic), steel	0.66/0.53 (manual/automatic) Mean	–	NR	Bimodal 0.66/0.68; 2.4/2.9 9.8/9.9; 1.7/1.7 (manual/automatic)	
Miettinen et al. (2016)	Welders; NR, GTAW/MIG, SS, general ventilation, NR	0.050 (middle of workshop) NR	0.088/0.14 × 10^6^ SD0.018/0.038 (BZ) 0.055/0.068 × 10^6^ SD 0.0061/0.017 (middle of workshop) mean	Multimodal: 0.010; 1.2, 0.016; 1.2, 0.027; 1.2	NR	Middle of workshop: unimodal with GMD of 0.046 µm, GSD 1.8
Sajedifar et al. (2018)	Experimental; Iran, SMAW, SS, no ventilation, NR	6.6 (23 cm) 3.8 (BZ/41 cm) mean	0.0038 × 10^6^ (23 cm) 0.0027 × 10^6^ (41 cm) mean	NR	NR	PM0.5: 44/21 num/wt%* PM1: 56/31 num/wt%* PM2: 56.4/37 num/wt%* (distance BZ/41 cm, for 23 cm see original article)
Yang et al. (2018)	Pipeline constructers; Taiwan, GTAW/SMAW, CS/SS, outdoors, NR	4.5 ± 0.31 Mean	–	NR	Bimodal 1.5; 3.2 19; 1.4	PM10: 40 wt%
Young et al. (2013)	Mixed welders; NR, spot welding, NR, general ventilation, activated-carbon facemasks (BZ)	0.10/0.19 (BZ) 0.092/0.14 (1.5 m) 0.11/0.12 (3–5 m) 0.12 (average) Mean	–	0.01–0.02	NR	PM0.1: 14 wt%* of PM4
Zugasti et al. (2012)	Apprentice welders; Spain, MAG/MMA, CS, general and local exhaust ventilation, NR	2.0–5.0 NR	–	NR	Bimodal 0.6/0.9; 2.0/2.2 7.9/8.6; 2.7/2.5 (MAG/MMA)	

The largest particle fraction is reported for mass concentration, for more details see supplementary table S2

*AM* arithmetic mean, *BZ* breathing zone, *CMD* count median diameter, *CPC* condensation particle counter, *CS* carbon steel, *FCAW* flux cored arc welding, *GM* geometric mean, *GMAW* gas metal arc welding, *GMD* geometric mean diameter, *GSD* geometric standard deviation, *GTAW* gas tungsten arc welding, *MAG* metal active gas, *MIG* metal inert gas, *MMA* manual metal arc welding, *MMAD* median mass aerodynamic diameter, *MS* mild steel, *NR* not reported, *PARPS* powered air purifying respirators, *PM* particulate matter, *PPE* personal protection equipment, *RT* real-time, *SD* standard deviation, *SMAW* shielded metal arc welding, *SMPS* scanning mobility particle sizer, *SS* stainless steel, *TIG* tungsten inert gas

*Calculated by us

### Reported size distribution of welding fume particles

The collected size distributions can be found in Table 1, or for more details in Table S1. Particle size distribution is usually defined in two categories; either based on geometric diameter or aerodynamic diameter. A geometric mean diameter (GMD) was reported or calculated in six studies, ranging between 0.01–0.10 μm out of which only one reported a multimodal distribution (Miettinen et al. 2016). Count median diameters (CMD) was reported in Lai et al. (2016) with a bimodal distribution with a 1st peak at 0.014–0.015 μm and a 2nd peak at 0.13–0.14 μm. Mass median aerodynamic diameter (MMAD) was further reported in four studies demonstrating a bimodal distribution with a 1st peak between 0.3–1.5 and 2nd peak at 1.4–9.8 μm. Debia et al. (2014) reported the main mode to range between 0.030–0.098 μm and 0.050–0.21 μm for different welding methods measured during the whole welding period or only during welding activities, respectively. Ham et al. (2012) reported the size distributions in a 3D-graph and therefore we could not process the data further.

Several studies reported size distribution in fractions, of which the smallest size fraction as well as size fractions closest to PM1, PM2.5 and PM10 are reported in Table 1. More details on the size fractional distribution can be found in Table S1. Based on particle number, the majority of the particles were reported to be below 0.1 μm in Debia et al. (2014) (48–88%), Graczyk et al. (2016) (92%), Ham et al. (2012) (64–68%) and Lehnert et al. (2012) (54%). Lai et al. (2016) and Young et al. (2013) reported 9 and 14% to be below 0.1 μm, respectively, based on mass. However, the mass-based majority of the particles were reported to be below 0.25 μm in Hedmer et al. (2014) (53%) and Lai et al. (2016) (84%).

The size-limit of the used instruments has a great impact on the final results of the size distributions and is an essential factor when comparing different studies, see details on the size-ranges of the various instruments in Table S1. The low end of the measurement range for the instruments varied from 0.0056 to 0.52 μm. The particles of interest in this study are primarily those within the nano-range (< 0.1 μm), therefore the studies investigating size distribution with a minimum size limit larger than 100 nm, including Kirichenko et al. (2019), Lin et al. (2015), Sajedifar et al. (2018), Yang et al. (2018), Zugasti et al. (2012), were not considered as central in the decision of the input values for the MPPD modelling.

### Reported exposure concentrations of welding fume particles

The reported exposure concentrations of welding fume particles from the 15 studies identified in the literature search are found in Table 1, for more details about methods and measurement position, see Table S2. A mixture of gravimetric and real-time measurements was identified where a majority of the studies measured total PM, while some performed only size-selective sampling including PM10, PM4, PM2.5 or ultrafine particles (for details see Table S2). Total particulate matter, inhalable or the largest reported size fraction concentrations are reported in Table 1, more details on other fractional concentrations can be found in Table S2.

A total of nine studies have reported mass concentrations of welding fumes specifically measured in the breathing zone of welders (see Table S2), ranging from 0.049 (PM2.5) to 45 (total PM) mg/m^3^. The majority of the breathing zone measurements were found in field studies reporting levels between 0.049 and 4.45 mg/m^3^, while the experimental reported 3.8–45 mg/m^3^. Measurements outside of the breathing zone, at various distances, ranged from 0.05 to 9 mg/m^3^. Gravimetrically measured mass concentrations ranged between 0.05 and 45 mg/m^3^, while real-time measurements ranged between 0.049 and 6.6 mg/m^3^. The overall concentrations of the lowest range were reported in the breathing zone by Lai et al. (2016) (0.049 mg/m^3^, PM2.5) as well as in the middle of the workshop by Miettinen et al. (2016) (0.05 mg/m^3^, total PM). The overall highest concentration was reported by Cena et al. (2016), measuring 45 mg/m^3^ at welders breathing zone. This was measured under experimental conditions inside a walk-in chamber under calm-air conditions representing welding in industrial facilities with no or poor ventilation or in restricted work environments with minimal air movements.

Number concentrations in the breathing zone ranged between 0.0027 and 2.7 × 10^6^ particles/cm^3^. The highest number concentration in breathing zone of 2.7 × 10^6^ particles/cm^3^ was reported by Cena et al. (2016) under conditions without ventilation. Measurements outside of the breathing zone at various distances from the source ranged between 0.00089 and 3.1 × 10^6^ particles/cm^3^. The overall highest number concentration was reported by Ham et al. (2012), reporting 3.1 × 10^6^ particles/cm^3^ in welding workplace with general ventilation through doors and windows. The overall lowest concentration was reported by Lai et al. (2016) in a semi-open welding area, however this only included particles above the size of 542 nm.

Due to different experimental designs and measurement approaches, it is generally difficult to draw any conclusions on concentration in breathing zone versus non-breathing zone, or gravimetric vs real-time measurements. Although some studies measured the concentration depending on specific welding parameters including welding method and base metal, this was outside the scope of this article and thus not distinguished in the tables.

### Justification for MPPD input values

Particle size distribution was reported in different formats including GMD and MMAD, of which a baseline size distribution value was selected based on the reported GMDs. Several studies reported a GMD of 0.041–0.069 μm, even though both larger and smaller GMDs were also reported (Table 1). The GMDs reported in the literature review are considered equal to count median diameter (CMD) if assuming log-normal distribution. Based on the reported size distribution, we selected a baseline particle CMD of 0.05 μm. Further, we assumed a single mode size distribution with a size geometric standard deviation (GSD) of 1.2. The complete baseline MPPD input values can be found in Table 2.

**Table 2:** MPPD input values—baseline

MPPD categories	Baseline input settings
Airway morphometry	human species; Yeh–Schum 5-lobe lung model; FRC = 3300 mL; URT volume = 50 mL
Particle properties	density = 7.9 g/cm^3^; aspect ratio = 1; count median diameter = 0.05 μm; inhalability adjustment checked; GSD (diam.) = 1.2
Exposure scenario: constant exposure	Acceleration of gravity = 981 cm/s^2^; body orientation = upright; aerosol concentration = 5 mg/m^3^; breathing frequency = 20/min; tidal volume = 1250 mL; inspiratory fraction = 0.5; breathing scenario = oronasal-mouth breather
	Number of hours per day = 6; number of days per week = 5; number of weeks = 1/45/2340; max. post-exposure days = 0
Clearance settings	tracheal mucous velocity = 5.5 mm/min; fast human clearance rate = 0.02/days, medium human clearance rate = 0.001/days; slow human clearance rate = 0.0001/day; lymph node human clearance rate = 0.00002/day

*FRC* functional residual capacity, *URT* upper respiratory tract

To determine the influence of size distribution on the deposition, the size ranges of CMD was selected between 0.01 and 1 μm with a GSD of 1.2 or 2, to cover all sizes reported in Table 1. To consider the reported aerodynamic diameters, MMAD between 0.35 and 1.4 μm with a GSD of 1.2 or 2 were also included.

The maximum and minimum reported exposure concentration was extracted directly from Table 1: 0.05 and 45 mg/m^3^.

### The deposition fraction depends on particle size, but distribution over generations is altered when normalizing for lung surface area

**Fig. 2:**
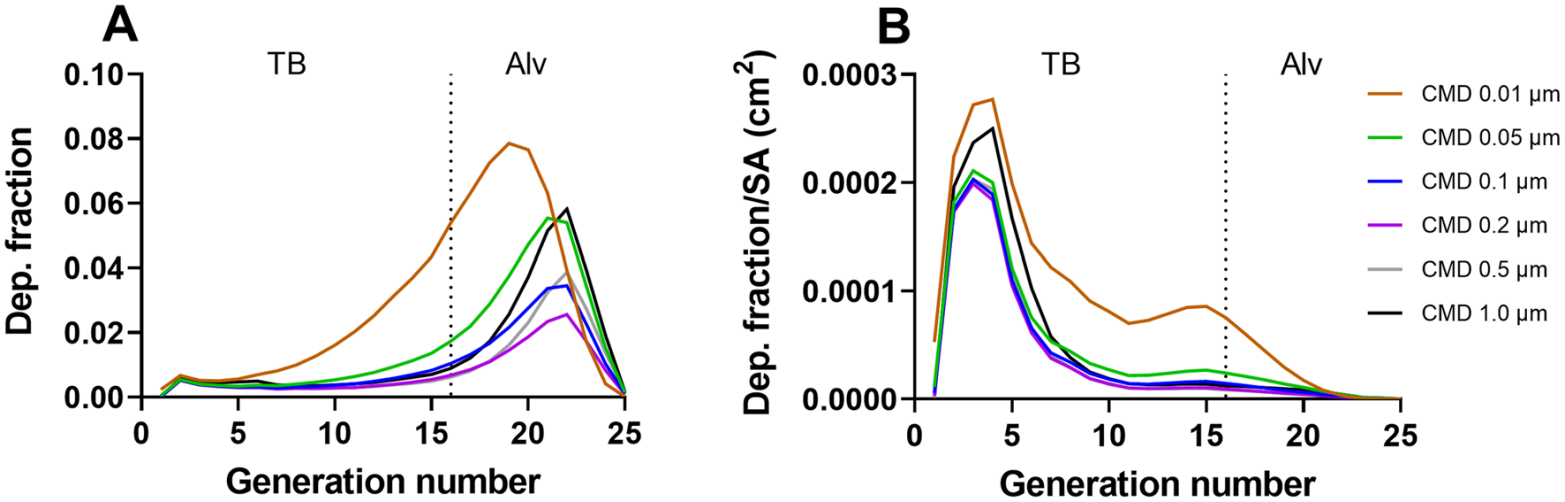
Deposition fraction per generation number depending on particle size distribution. Results are expressed as deposition fraction over generation number (**A**) or deposition fraction per surface area (SA) over generation number (**B**). Baseline input values were used including the occupational exposure limit concentration of 5 mg/m^3^ and moderate workload. The size distributions were varied from CMD 0.01–1 μm with a of GSD 1.2

The impact of particle size distribution on the regional deposition fraction during a single breath was determined using a variation of size distributions of CMD ranging from 0.01 to 1 μm as compared to baseline input values (CMD 0.05 μm). The results expressed in deposition fraction over generation number are visualized in Fig. 2A. Overall, the smallest size of CMD 0.01 µm resulted in the largest total deposition. In the tracheobronchial region, CMD 0.01 μm was deposited to the largest extent, followed by CMD 0.1 μm. The remaining sizes were found to deposit in the tracheobronchial region to a similar extent. In the alveolar region, CMD of 0.01 μm resulted in the largest deposition, followed by 0.05 and 1 μm. CMD 0.2 μm was deposited to the least extent in the alveolar region.

Figure 2B shows the deposition fraction normalized to regional lung surface area (deposition fraction/cm^2^) over generation number. As a result of the varying surface areas of the different region of the lung, the deposition distribution over generation number is shifted, as compared to Fig. 2A. The results demonstrate an overall higher deposition fraction per surface area in the tracheobronchial region, particularly in the first generations, as compared to the alveolar region.

Identical simulations were performed with MMAD as size distribution input (MMAD 0.35–1.4 μm, GSD 1.2), Figure S1B. The results on deposition fraction demonstrate the smallest MMAD of 0.35 μm to result in the highest total tracheobronchial and alveolar deposition. A size distribution with a MMAD of 0.7 μm were found to deposit the least in the alveolar region but comparable to MMAD 1.4 μm in the tracheobronchial region. When normalizing for surface area, similar to what was observed for GMD, the distribution was shifted to result in overall higher deposition in the upper generation numbers. The same simulations (CMD 0.01–1 μm/MMAD 0.35–1.4 μm) were performed with a GSD of 2 (Figure S1A, C), but no substantial effect was seen on the results.

### Lung retention depends largely on particle size, concentration, and workload

**Fig. 3:**
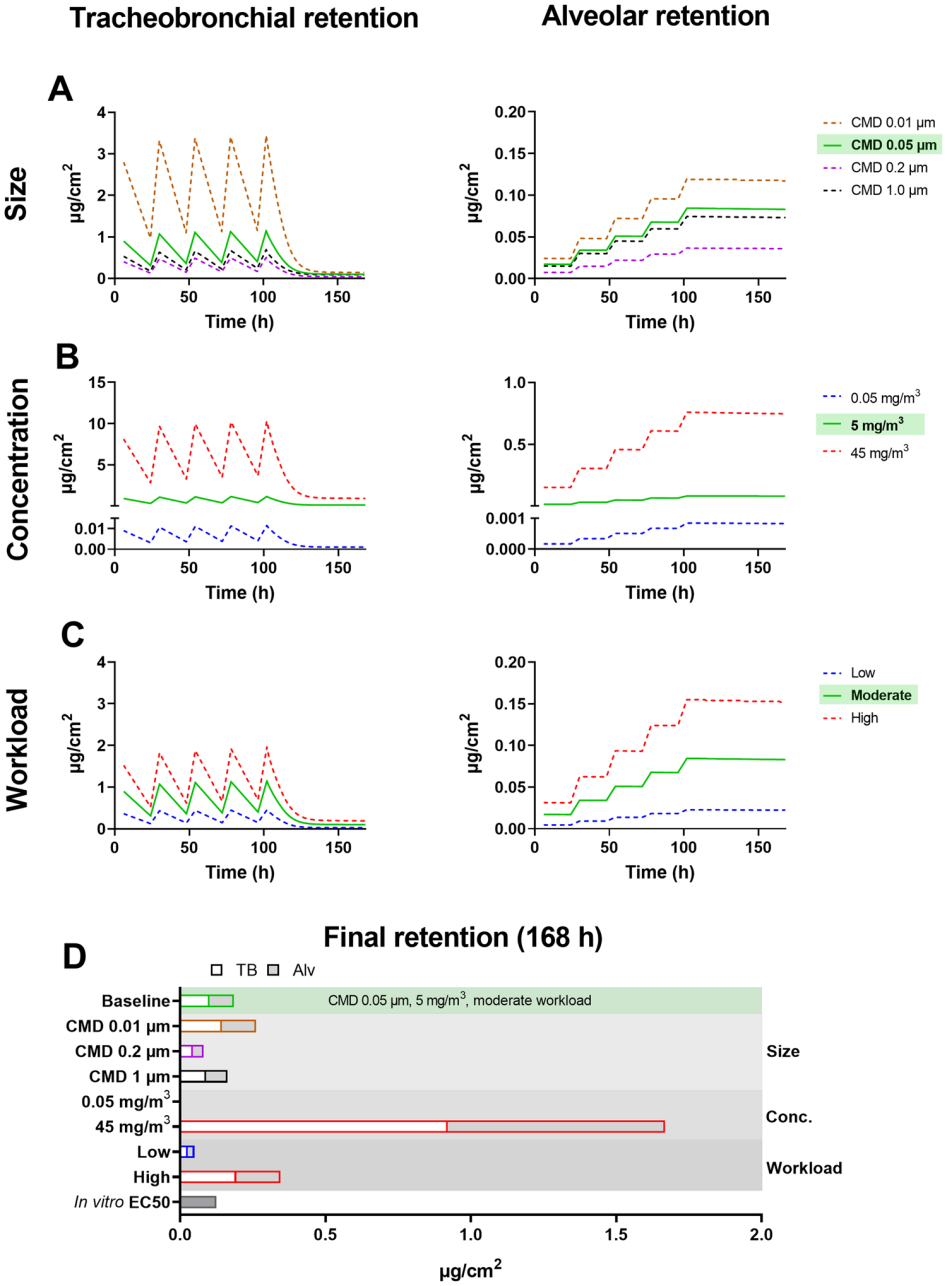
MPPD model results of tracheobronchial and alveolar retention per surface area versus time (h) during 1-week simulation assuming 6 h of exposure the first 5 days, followed by 2 days of only clearance. Baseline input values are marked in green and include a CMD of 0.05 μm (GSD 1.2), the occupational exposure limit concentration of 5 mg/m^3^ and a moderate workload. The influence of a variation of **A** size distribution (GSD 1.2), **B** concentration and **C** workload (breathing pattern) were further explored. Final tracheobronchial and alveolar retention after 1 week (incl. weekend) for all simulations are shown in **D**. The in vitro EC50 included in **D** represents a responsive cell dose found to elicit a reduction of 50% cell viability in human bronchial epithelial cells following 24-h exposure

The regional lung retention (deposition and clearance) of welding fume particles was determined following a simulation of 1 week (5 days exposure, 6 h/day followed by 2 days of no exposure) for baseline input values as well as a variation of size distribution, exposure concentration, workload (Fig. 3), density and body position (Figure S2). In agreement with Fig. 2, the deposition in the tracheobronchial region is generally larger compared to the alveolar region but the results demonstrate that the clearance mechanisms in the tracheobronchial region are more effective resulting in a rapid decrease in retention following time of non-exposure. In contrast, the retention of the alveolar region, which has much slower clearance mechanisms, is instead slowly built up over time. At the end of the work shift of day 1 or day 5, with baseline values, the tracheobronchial retention corresponded to 0.89 and 1.15 μg/cm^2^ with an alveolar retention of 0.017 and 0.085 μg/cm^2^, respectively. At the end of the 1-week simulation, including 2 days without exposure and only clearance, the retention corresponded to 0.102 and 0.083 μg/cm^2^ in the tracheobronchial and alveolar region, respectively. The following results reflect the retention at the end of 1 week.

When altering the size distribution of the welding fume particles, the tracheobronchial and alveolar retention was increased 1.4-fold by CMD 0.01 μm compared to CMD 0.05 μm (baseline). In contrast, a CMD of 1 and 0.2 μm resulted in a decrease of 1.1- or 2.3-fold, respectively.

The alveolar and tracheobronchial retention was found to change linearly as the external exposure concentration was altered. The maximum concentration of 45 mg/m^3^ resulted in a tracheobronchial and alveolar retention of 0.92 and 0.75 μg/cm^2^, corresponding to a ninefold increase compared to baseline concentration of 5 mg/m^3^. The minimum concentration of 0.05 mg/m^3^ resulted in a retention of 0.001 and 0.0008 μg/cm^2^ in the tracheobronchial and alveolar region, respectively, corresponding to more than a 100-fold decrease from the baseline value.

An increase in workload from medium (baseline) to high resulted in a 1.9- and 1.8-fold increase in the retention in the tracheobronchial and alveolar region, respectively. The retention following low workload resulted in 0.027 and 0.022 μg/cm^2^ in the tracheobronchial and alveolar region, respectively, corresponding to an approximately fourfold decrease compared to baseline retention.

As visualized in Fig. 3D, our results demonstrate that aerosol concentration is the most important input parameter followed by workload and size distribution. In the 1-week simulation varying density and body position, no effect on the retention was observed (Figure S2).

### Alveolar retention is larger than tracheobronchial retention following chronic exposure

**Fig. 4:**
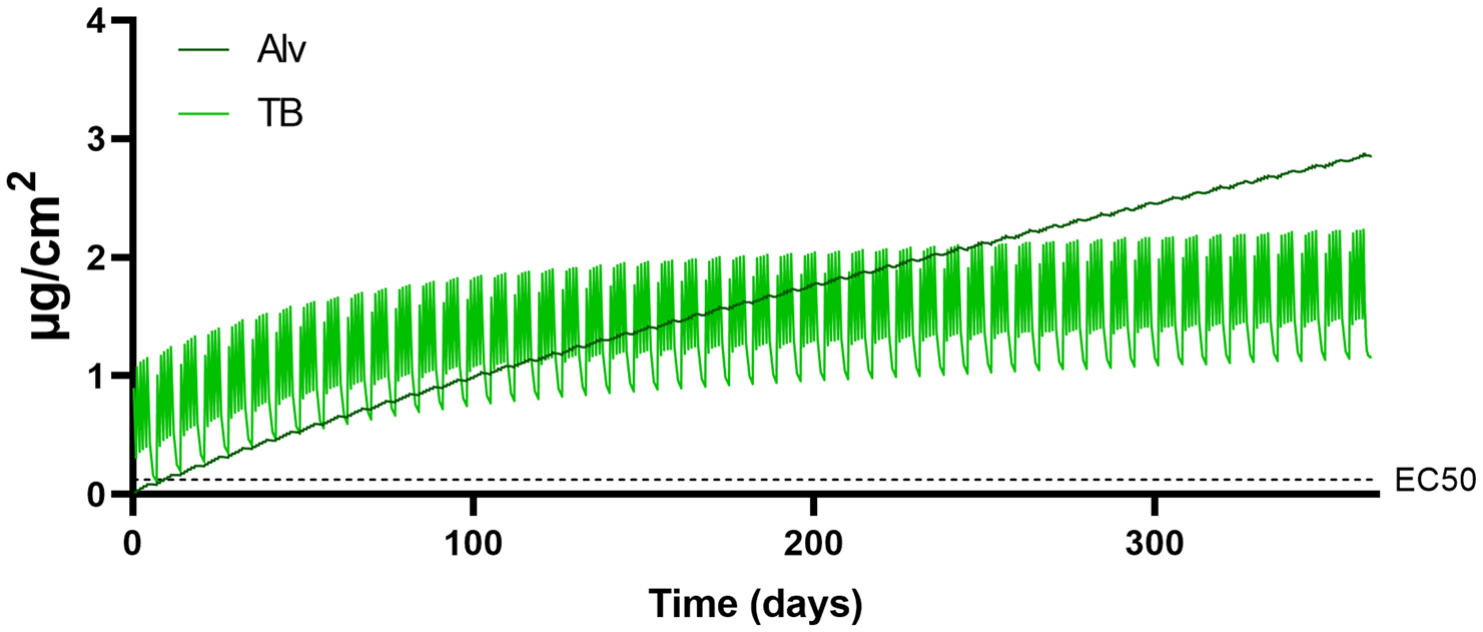
MPPD model results of tracheobronchial or alveolar welding fume mass retained per surface area versus days of exposure up to 1 year. Baseline input values were used including the occupational exposure limit concentration of 5 mg/m^3^, a size distribution of CMD 0.05 μm, GMD 1.2 and a moderate workload. The in vitro EC50 represents a responsive cell dose found to elicit a reduction of 50% cell viability in human bronchial epithelial cells following 24-h exposure

**Fig. 5:**
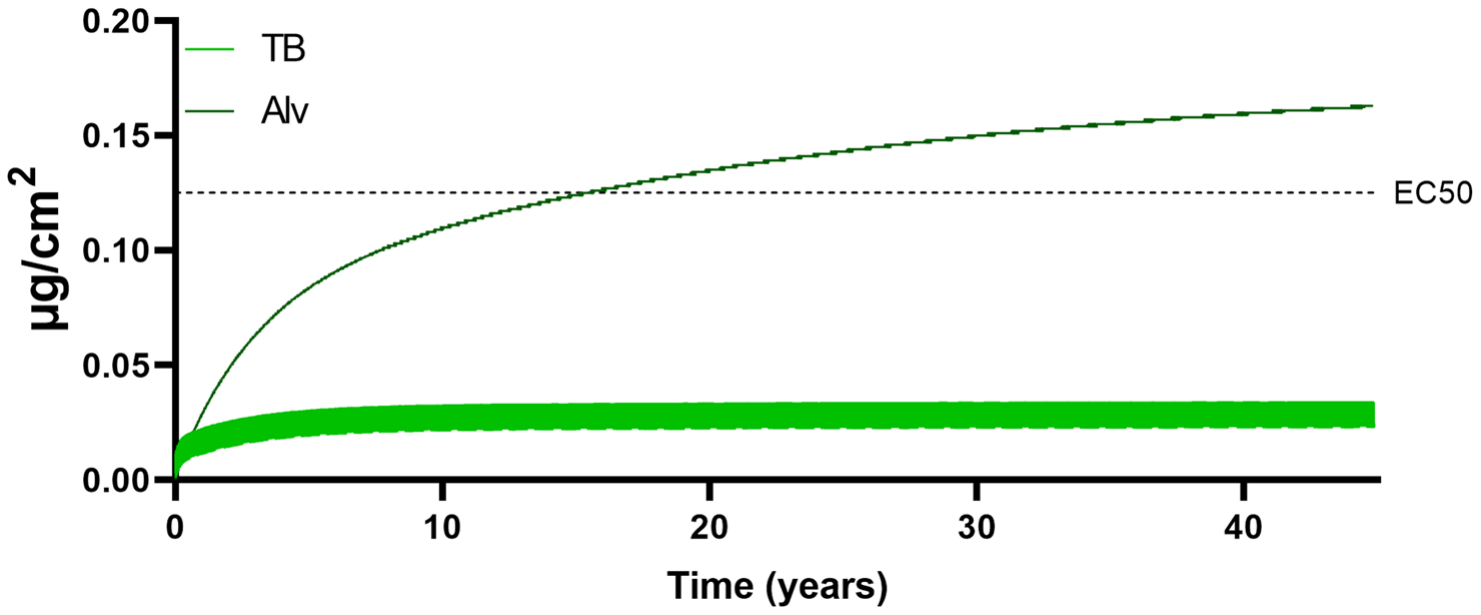
MPPD model results of tracheobronchial or alveolar welding fume mass retained per surface area versus years of exposure up to 45 years. An occupational exposure of 0.05 mg/m^3^ was used with remaining baseline input values including a size distribution of CMD 0.05 μm, GSD 1.2 and a moderate workload. The in vitro EC50 represents a responsive cell dose found to elicit a reduction of 50% cell viability in human bronchial epithelial cells following 24-h exposure

The alveolar and tracheobronchial retention during 1-year simulation using the baseline input values is visualized in Fig. 4, while a life-time occupational exposure (45 years) at the low-end exposure concentration of 0.05 mg/m^3^ is visualized in Fig. 5. The results confirm the build-up of alveolar retention, while the tracheobronchial region levels out to a larger degree. In the 1-year simulation with baseline input, the alveolar retention is found to exceed the tracheobronchial retention after approximately 250 days. At the end of 1 year, the tracheobronchial and alveolar retention was 1.15 and 2.85 µg/cm^3^, respectively, corresponding to a 11- and 34-fold increase from the 1-week exposure. After 45 years exposure to 0.05 mg/m^3^, the tracheobronchial and alveolar retention corresponded to 0.023 and 0.16 µg/cm^2^.

### Comparison between modelled lung doses and those used in vitro

To enable the comparison of real lung doses to those used in vitro, the estimated lung dose (retention) following the different exposure scenarios (altering concentrations and time of exposure) are compiled in Table 3, together with in vitro doses as extracted from McCarrick et al (2021) corresponding to 1.6–31.3 µg/cm^2^ when considering the nominal dose, or 0.08–1.57 µg/cm^2^ when considering cell dose (assuming 5% uptake). One-year exposure to the OEL-level of 5 mg/m^3^ resulted in an alveolar retention exceeding the entire cell dose range tested in vitro.

**Table 3:** Deposited welding fume particle dose for human inhalation exposure and in vitro exposure systems

Exposure scenario	Exposure concentration	Exposure duration	Dose (µg/cm^2^)	
Human inhalation			TB	Alv
OEL exposure	5 mg/m^3^	6 h	0.89	0.017
		1 week	0.102	0.083
		1 year	1.15	2.85
Low end exposure	0.05 mg/m^3^	Lifetime (45 years)	0.023	0.16
High end exposure	45 mg/m^3^	1 week	0.92	0.75
In vitro (HBEC-3kt)			Nominal	Cell dose
Dose range	5–100 µg/mL	24 h	1.6–31.3	0.08–1.57
EC50	8 µg/mL		2.5	0.125

The modelled lung doses are presented in the tracheobronchial (TB) or alveolar (Alv) region. For the in vitro doses we assume 100 µL of nanoparticle suspension is given into one well of a standard 96-well plate with a growth area of 0.32 cm^2^ per well assuming full sedimentation and bioavailability (nominal) or with a 5% uptake (cell dose). The in vitro EC50 corresponds to the responsive cell dose of the most toxic welding fume found to elicit a reduction of 50% cell viability in human bronchial epithelial cells following 24-h exposure tested in McCarrick et al. (2021)

The cytotoxic in vitro EC50 cell dose of 0.125 µg/cm^2^ was reached and exceeded in the tracheobronchial region following 6 h exposure to the OEL 5 mg/m^3^ (sevenfold). However, this was not the case at the end of 1 working week including weekend, i.e., 2 final days of only clearance and no exposure. The EC50 was further exceeded in both the tracheobronchial and alveolar region following 1 year of exposure to OEL 5 mg/m^3^ (9.2-fold and 23-fold in tracheobronchial and alveolar region, respectively) or 1 week of exposure to the high-end concentration of 45 mg/m^3^ (seven and sixfold in tracheobronchial and alveolar region, respectively). In addition, an occupational life-time exposure to the low-end concentration of 0.05 mg/m^3^ resulted in alveolar retention comparable to the EC50 cell dose.

The EC50 was determined in bronchial epithelial cells, however we assume similar potency in alveolar cells.

## Discussion

The purpose of this study was to investigate the deposition of welding fume particles in the different regions of the lung to facilitate the translation between human exposure and in vitro concentrations to ultimately improve risk characterization. Using the MPPD model, we simulated various real-life occupational exposure scenarios to welding fumes under short- and long-term (6 h up to 45 years). Our results imply that the lung dose following these exposure scenarios to welding fumes are within the range of primarily low-end cell dose concentrations currently used in vitro. Nonetheless, our results further show that a 1-year occupational exposure to the OEL of 5 mg/m^3^ results in alveolar retention exceeding the cell dose following the highest concentration (100 µg/mL) tested in vitro. Interestingly, real-life occupational lung doses over both short and long-term exposure were found comparable to cell doses where toxic effects have been observed in vitro using human bronchial epithelial cells. Important to note though is that the in vitro effective dose is based on a bolus dose assessed at the acute timepoint of 24 h, while corresponding lung doses are attained primarily following exposure over longer duration. However, the in vitro EC50 dose was found to be exceeded in the tracheobronchial region already after 1 working day (6-h exposure) at OEL aerosol concentration. The results demonstrate the potential of using real-life exposure data in combination with particle deposition modelling to improve the internal lung dose estimates. This will enhance the understanding of in vitro concentrations in the context of human occupational exposure and can be used as a tool in the study design of in vitro studies.

Our results emphasize the short-term concern for the tracheobronchial region but primarily the concern of alveolar retention for long-term occupational exposures to welding fume particles. The MPPD model predicts uniform deposition in each lung region meaning that all cells receive the same average dose. However, the deposition of particles in a human lung may be more heterogenous and contain hotspots, i.e., certain sites that may accumulate large fractions of the particles deposited. As an example, Ishikawa et al. (1994) demonstrated chromium concentrations to be higher at airway bifurcations than in neighboring epithelial tissue in exchromate workers. Based on this, our results are likely an underestimation of the tracheobronchial doses for cells located in certain areas, such as bifurcations. To further refine the deposition pattern derived from MPPD, complimentary models such as computational fluid dynamics models (CFD) should be considered to predict local deposition. By CFD modelling, Balashazy et al. (2003) demonstrated an enhancement of the deposition at tracheobronchial bifurcations to more than 100 times for micron-sized particles, and 5 to 60-fold for nanosized particles as compared to the average values. Using this data, Phalen et al. (2006) further compared the tracheobronchial deposition by MPPD modelling with or without the addition of CFD-data for justification of in vitro doses, and demonstrated a 100 times difference in surface particle deposition. This suggest that if we would, as an example, include a safety factor of 100 for hotspots, the tracheobronchial retention following a lifetime exposure to low-end concentrations would increase from 0.023 to 2.3 µg/cm^2^, and would thus exceed the entire range of cell doses tested in vitro. This would also alter the relationship between tracheobronchial and alveolar retention, where the tracheobronchial retention would exceed the alveolar retention in many cases.

The balance between deposition and clearance determines particle retention and lung burden. Default clearance settings of the MPPD model, as was used in this study, assume poorly soluble particles (Miller et al. 2016), whereas welding fume particles are expected to dissolve to varying extent (Antonini et al. 1999, 2005; McNeilly et al. 2004; Berlinger et al. 2019; McCarrick et al. 2019). The use of animal models could provide valuable mechanistic understanding of the fate of particles in an intact organism. Animal studies have demonstrated that various metals found in welding fumes are cleared from the lung at substantially different rates (Lam et al. 1978; Antonini et al. 2010, 2011). For example, whereas more than 80% of total deposited Mn had been cleared by 8 days and nearly all Mn by 21 days (in rats exposed to mild steel fume via inhalation), significant amounts (around 40%) of Fe remained in the lungs at 42 days after exposure (Antonini et al. 2011). Based on this together with findings of an initial quick clearance face of Fe, welding fume clearance has been suggested to be initially governed by mucociliary and lung macrophage clearance, followed by dissolution over time. The inclusion of welding-specific clearance and dissolution kinetics would reduce the residence time of the particles and thus the retention, especially in the long-term simulations. By including the kinetics of welding particle dissolution, this could also give valuable estimates of the internal lung dose of metal ions being released from welding fume particles. The MPPD model has previously been used to correlate lung deposition of silver nanoparticles to reported in vitro toxic threshold of ionic silver by assuming dissolution to a certain extent (Smith and Skinner 2021). The release of hexavalent chromium has for instance been suggested to be largely involved in welding particle toxicity by us (McCarrick et al. 2019, 2021) and others (Antonini et al. 1999, 2005; McNeilly et al. 2004), therefore this approach may be useful also for welding induced toxicity. To further refine dose estimates, MPPD models can also be combined with PBPK models, which has been carried out to derive target dose of manganese in human brain after inhalation of welding fumes (Ramoju et al. 2017).

The exposure concentration was found to be the most influential input parameter for lung retention of welding fume particles in our study, followed by workload and particle size distribution. The majority of the reported mass concentrations were below the OEL for inorganic inhalable dust of 5 mg/m^3^ but the levels varied to a large extent. The levels were, however, well in accordance with the welding fume concentrations reported by IARC ranging from < 1 to 25 mg/m^3^ for stainless steel, and < 1 to > 50 mg/m^3^ for mild steel (IARC 2017). This emphasizes the need to minimize exposure concentration, primarily with secondary protection such as proper ventilation and personal protection equipment. Factors in the concept of workload included breathing frequency and tidal volume, where an increased flow is expected to enhance deposition. Workload has been shown to vary among welders, where the majority were assessed as working under medium physical workload (Lehnert et al. 2015). Our results are in line with Gangwal et al. (2011), who also identified aerosol concentration and breathing patterns as primary key determinants in the prediction of alveolar retention of various metal-based nanoparticles in a sensitivity analysis using the MPPD model.

It is well established that particle size is a predictor of the site of deposition in the respiratory tract (Oberdörster et al. 2005; Hofmann 2020). The literature compiled suggest that welding fumes contain a high proportion of nanosized particles. However, the variation in reported size distribution is likely a reflection of altered factors such as welding process, wire feed rate and emission rate (Brand et al. 2012; Ennan et al. 2013). Our results confirmed the deposition of welding fume particles to be dependent on particle size distribution, which emphasizes the importance of well characterized welding fumes and the use of case-specific size distributions in deposition assessments. This can be exemplified by comparing results of our study with deposition fraction used by Falcone et al. (2017) in their human relevance deposition calculation. They used a deposition fraction of 0.16 for welding particles with a reported MMAD of 0.31 µm, whereas our baseline total deposition fraction was 0.43 for CMD of 0.05 µm. Moreover, the size of the particles may further influence lung retention in other ways than altering deposition. For example, particle size can impact clearance efficiency, where smaller nanoparticles have been suggested to escape phagocytosis to a greater extent compared to larger sized particles (Kreyling et al. 2006; Yang et al. 2008).

The in vitro doses used in the comparison were employed under submerged conditions for human bronchial epithelial cells (HBEC-3kt) (McCarrick et al. 2021). Other in vitro studies investigating welding fume toxicity have applied nominal doses varying between 2 and 250 μg/mL with a lowest dose generally ranging from 2 to 6.25 μg/mL (Antonini et al. 1999, 2005; McNeilly et al. 2004; Leonard et al. 2010; McCarrick et al. 2019; Hedberg et al. 2021). However, nominal media concentrations have been argued to be considered as measures of exposures rather than dose due to that they do not accurately reflect the dose in contact with the cells (Teeguarden et al. 2007; Groothuis et al. 2015). Therefore, the in vitro cellular dose, and not the nominal dose, was primarily considered in the comparison to the lung retention of welding fumes, demonstrating that response in human bronchial epithelial cells can be detected at cell doses attained following real-life exposure scenarios. The cellular dose was based on the 5% uptake as measured by ICP-MS in McCarrick et al. (2021), which can be considered as relatively low uptake. However, even if assuming 50% uptake corresponding to a EC50 cell dose of 1.25 μg/cm^2^, this is still within a comparable magnitude as the modeled lung doses.

The in vitro EC50 was based on the most cytotoxic welding fume tested at 24 h and can therefore be considered somewhat a worst-case exposure, whereas other welding fume particles elicited a cytotoxic response first when high-end concentrations were used (McCarrick et al. 2021). In a recent study by Samulin Erdem et al. (2020), mild steel welding fumes were tested in vitro at low concentrations ranging between 0.035 and 4.375 μg/mL for 6 h per day during 5 consecutive days to more closely mimic occupational exposure patterns. However, the results demonstrated no cytotoxic effects on macrophages, epithelial or endothelial cells. Some studies have suggested that a higher threshold is required to elicit effects to occur in vitro compared to in vivo (animals). As an example, pro-inflammatory effects of poorly soluble nanomaterials were demonstrated to occur at lower alveolar surface doses in vivo compared to in vitro, where in vitro effects were observed at doses ten times higher at ALI or 20–100 times higher at submerged exposure (Loret et al. 2018). The higher concentrations required for in vitro response is pointing towards the higher complexity of in vivo models compared to the relatively simple in vitro models and could thus motivate the use of higher dose levels in vitro. Further research is however needed to find appropriate converting factors between in vitro and in vivo effects. More advanced cellular models including co-cultures or 3D-cultures as well as exposure under air–liquid interface (ALI) should also be considered in the future.

To our knowledge, this is the first study to model lung deposition of welding fume particles following simulations of real-life exposure scenarios and relate them to in vitro dose levels. As emphasized in Phalen et al. (2021), the experimental design of in vitro studies should be aiming at predicting effects in the target tissue of humans for specific use cases, including specific size distributions and ventilation rates relevant to the target population of interest, in which MPPD modelling offers this possibility. However, to facilitate the usage of dosimetry models, relevant and accurate exposure and particle characteristics data need to be available. In general, most studies investigating welding fume exposure did not measure or report size distribution, resulting in 64 out of the 79 articles identified being excluded due to insufficient size distribution data. To improve the deposition modelling of welding fumes, future studies should include measurements on both particle size and concentration. Another important aspect to highlight is the diversity in study design, measurement approaches and reporting of results of the different studies reviewed, probably a result of the miscellaneous aims of the studies. To obtain comparable and useful data for dosimetry modelling, harmonization of the measurement instruments and strategies would be beneficial.

## Conclusion

The results reveal the concern of primarily tracheobronchial retention for short-term exposure to welding fumes, whereas alveolar retention is built up over time and thus of more concern for chronic exposure. Our results further suggest that lung doses retained following real-life occupational exposure scenarios of welders can be compared to cell doses found to elicit a toxic effect in vitro. The large deposition fraction in the tracheobronchial region results in retention exceeding the in vitro toxic dose already after one working shift at occupational exposure limit-level, although the retention is rapidly decreased after exposure stops. The regional lung deposition depends largely on aerosol concentration and time of exposure where the low-end exposure levels resulted in alveolar retention comparable to toxic in vitro doses first after 15–20 years and the tracheobronchial retention only if considering hotspots. The influence of parameters including aerosol concentration, size distribution and workload for lung retention was demonstrated, which emphasizes the importance in selection of input values specific to the target population. In all, this study demonstrates the potential of combining real-life exposure data with particle deposition modelling to improve lung dose estimates and provide a tool for dose selection in vitro. Understanding the association between occupational exposures and in vitro doses would improve study design as well as interpretation of toxicological results and hazard assessments.

## Supplementary Information

Below is the link to the electronic supplementary material.

Supplementary file1 (DOCX 608 KB)
