# Supplementary material for: Modelled lung deposition and retention of welding fume particles in occupational scenarios: a comparison to doses used in vitro
Source: Arch Toxicol. 2022 Feb 21;96(4):969–85. doi: 10.1007/s00204-022-03247-9 (PMC8921161; doi:10.1007/s00204-022-03247-9)
Supplement: Supplementary file 1 — Supplementary file1 (DOCX 608 KB) [file 204_2022_3247_MOESM1_ESM.docx]

# Supplementary tables and figures

## Modelled lung deposition and retention of welding fume particles in occupational scenarios – a comparison to doses used in vitro

Sarah McCarrick^1*^, Hanna L. Karlsson^1^, Ulrika Carlander^1^

^1^Institute of Environmental Medicine, Karolinska Institutet, 171 77, Stockholm, Sweden.

^*^corresponding author: sarah.mccarrick@ki.se

**Table S1.** Summary welding methods, conditions and size distribution in the literature compilation.

Abbreviations: Al= aluminium, APS= aerodynamic particle sizer, BZ= breathing zone, CMD= count median diameter, CS= carbon steel, ELPI= electrical low pressure impactor, FCAW= flux cored arc welding, FMPS= fast mobility particle sizer, GMAW= gas metal arc welding, GMD= geometric mean diameter, GSD= geometric standard deviation, GTAW= gas tungsten arc welding, MAG= metal active gas, MIG= metal inert gas, MMA= manual metal arc/, MMAD= median mass aerodynamic diameter, MS= mild steel, NR= not reported, PM= particulate matter, SMAW= shielded metal arc welding, SMPS= sequential mobility particle sizer, SS= stainless steel.

| Reference | Population; Location, Welding method, base material | GMD (µm); GSD | MMAD (µm);  GSD | Size distribution instrument (metric; size range ) | Comments/additional data |
| --- | --- | --- | --- | --- | --- |
| Cena (2016) | Experimental; NR, GMAW, MS | 0.20* | 0.35; 1.5 (BZ)  0.30; 1.4 (2 m) | Deposition impactor MOUDI 10/115(mass; 15 fractions 0.01-32 µm)  SMPS TSI 3080 (number; 0.01-0.4)  APS TSI 3321 (number; 0.5-10 µm) | Bimodal distribution with small second peak around 0.01 µm |
| Debia (2014) | Apprentice welders; Canada, FCAW/GMAW/GTAW/SMAW, Al/steel/SS | NR | NR | ELPI, Dekati (number; 12 stages 0.007-10 µm ) | Ultrafine particles (<0.1 µm): 48-88 % (welding activity), 75-93 % (whole welding period)  Main modes around:  50/214/98/50 nm (welding activity), 30/30/98/30 nm (whole welding period) for FCAW/GMAW/GTAW/SMAW |
| Graczyk (2016) | Apprentice welders; Switzerland, TIG, Al | 0.045; 1.6 (BZ)  0.051; 1.5 (0.6 m, DiscMini)  0.069; 1.9 (0.6 m, SMPS) | NR | DiscMini Matter Aeorosol (number; 0.8-µm cutoff)  SMPS Grimm 55-40-25 DSA, 5.403 CPC (number; 0.01-1.11 µm) | <0.1 µm: 92 num%  <0.041 µm: 50 num% |
| Ham (2012) | Welders; NR, GMAW, MS | NR | NR | SMPS TSI 3936L75 (number; 0.015-0.661 µm) | 3D-format graph: no data extracted  <0.1 µm: 64-68 num%* |
| Hedmer (2014) | Welders in manufacturing; Sweden, GMAW/MIG/MAG, MS | NR | NR | Personal cascade impactor, Sioutas SKC (mass; 5 stages) | <0.25 µm: 53 wt%,  0.25-0.5 µm: 11 wt%,  0.5-1 µm: 9 wt%,  1-2.5 µm: 10 wt%,  2.5-10 µm: 18 wt% |
| Insley (2019) | Welders in metal products fabrication; Pennsylvania US, FCAW, CS | NR | NR | DustTrak TSI DRX 8533 (mass; 0.1-15 µm) | <1 µm: 80 wt%* ,  <2.5 µm 80 wt%* ,  <4 µm 83 wt%* ,  <10 µm 92 wt%* |
| Kirichenko (2019) | Experimental, NR, Arc welding, Low alloyed steel | NR | NR | AeroTrak handheld particle counter TSI 9306 (number; NR) | <0.3 µm: 78-83 num%*,  0.3-0.5 µm: 16-20 num%*,  0.5-1 µm: 1.1-1.7 num%*,  1-3 µm: 0.07-0.15 num%*,  3-5 µm: 0.02-0.06 num%*,  5-10 µm: 0.002-0.01 num%*  at height 0.8 m from source and 15 min settling time |
| Lai (2016) | Shipyard welders; Taiwan, TIG, Galvanized metal | 0.041* | NR | SMPS TSI 3936 (number; 0.005-0.16 µm)  APS TSI 3321 (number; 0.542-19.81 µm)  MOUDI, MPS (mass; 11 stages 0.056-18 µm) | CMD: Bimodal   - 0.014-0.015 µm - 0.13-0.14 µm   PM2.5: 84 wt% of PM10  PM0.1: 9 wt% of PM10* |
| Lehnert (2012) | Mixed welders; Germany, FCAW/GMAW /SMAW/TIG  /Miscellaneous, SS/MS/other | FCAW: 0.10*  GMAW: 0.090*  SMAW: 0.067*  TIG: 0.042* | NR | SMPS TSI (number; 0.014-0.673 µm) | <0.1 μm: 54 num%* |
| Lin (2015) | Fitness equipment manufacturers; Taiwan, NR, steel | NR | Bimodal  0.66/0.68; 2.4/2.9  9.8/9.9; 1.7/1.7  (manual/automatic) | Cascade impactor Marple 225-50-001 (mass; 8 stages) | Manual and automatic welding. |
| Miettinen (2016) | Welders; NR, GTAW/MIG, SS | Multimodal:  0.010; 1.2,  0.016; 1.2,  0.027; 1.2 | NR | FMPS TSI 3091 (number; 0.0056-0.56 µm) | Middle of workshop: unimodal with GMD of 0.046 µm, GSD 1.8 |
| Sajedifar (2018) | Experimental; Iran, SMAW, SS | NR | NR | Dust monitor GRIMM 1.106 (number and mass, 8 stages 0.35- >6.5 µm) | 0.35-0.5: 44/21 num/wt%*,  0.5 -0.75: 11/7 num/wt%*,  0.75-1: 1.1/4 num/wt%*,  1-2: 0.3/6 num/wt%*,  2-3.5: 0.1/8 num/wt%*,  3.5-5: 0.1/10 num/wt%*,  5-6.5: 0.02/6 num/wt%*,  >6.5: 0.03/18 num/wt%*  (distance 41 cm (BZ), for 23 cm see original article) |
| Yang (2018) | Pipeline constructers; Taiwan, GTAW/SMAW, CS/SS | NR | 1.5; 3.2 (< 10 µm)  19; 1.4 (>10 µm) | Cascade impactor Marple 298 (mass; 8 stages 0.1- 31.35 µm) | <10 µm: 40 wt%  >10 µm: 60 wt% |
| Young (2013) | Welders; NR, spot welding, NR | 0.01-0.02 | NR | SMPS GRIMM 5.500 (number; 0.0055-0.350 µm) | PM0.1: 14 wt%*  PM0.1-4: 86 wt%*  (based on average of distances, for specific distances see original article) |
| Zugasti (2012) | Apprentice welders; Spain, MAG/MMA, CS | NR | Bimodal  0.6/0.9; 2.0/2.2  7.9/8.6; 2.7/2.5  (MAG/MMA) | Cascade impactor Marple 298 (mass; 8 stages) |  |

* calculated by us.

**Table S2.** Exposure levels quantified in the literature compilation. Exposure level expressed as arithmetic mean unless indicated otherwise. Note: ^a^GSD. Abbrevations: AM= arithmetic mean, BZ= breathing zone, CI= cascade impactor, CPC= condensation particle counter, FMPS= fast nobility particle sizer, GM= geometric mean, GSD= geometric standard deviation, GV= general ventilation including natural ventilation, LEV = local exhaust ventilation, LOD= limit of detection, NR= not reported, PARPS= powered air-purifying respirators, PENS= personal nanoparticle sampler, PM= particulate matter, PPE= personal protective equipment, PS= personal sampler, PTFE= polytetrafluoroethylene, PVC= polyvinyl chloride, RT= real time, SD= standard deviation, SMPS= sequential mobility particle sizer, UFP= ultrafine particles

| Reference | mg/m^3^± SD | Particles/cm^3^; GSD | Method (instrument): sampling matrix (fraction): distance | Comments/additional data |
| --- | --- | --- | --- | --- |
| Cena (2016) | 45 ± 2.2 (BZ)  9 ± 2.2 (2 m)  mean | 2.7 x 10^6^ (BZ)  2.6x 10^6^ (2 m)  NR | Gravimetric (PFTE filter, SKC PCXR4): mass (total): BZ/2 m  Real time (CPC, TSI 3007): number: BZ/2m | 2.5x3.5x2.7 m area with no ventilation |
| Debia (2014) | - | 0.065-0.17x10^6^; 1.2-1.7  GM | Real time (P-Trak 8525 CPC, 0.02-1 µm): number (total): near BZ | General and local exhaust ventilation |
| Graczyk (2016) | 0.72 (BZ)  0.67 (0.6 m)  median | 1.7x10^6^; 2.4(BZ)  1.1x10^6^ ;1.9 (0.6 m, DiscMini)  0.77x10^6^; 1.6 (0.6 m, SMPS)  GM | Gravimetric (PFTE filter, Parallel particle impactor, PPI SKC): mass (PM4): BZ/0.6 m  Real time (DiscMini particle counter, < 0.8 µm): number (total): BZ/0.6 m  Real time (SMPS Grimm 55-40-25 DMA, 5.403 CPC, 0.01-1.11 µm): number (total): 0.6 m | 10 m^3^  area with pulsing ventilation  PPE: Non-ventilated helmet  BZ – inside helmet |
| Ham (2012) | 0.20/0.35 (PM1)  0.48/1.1 (total)  GM | 0.2/2.1 x10^6^; 2.7/2.9 (<0.1 µm)  0.31/3.1x10^6^; 2.7/3.1 (SMPS total)  0.034/0.054x10^6^ (CPC total)  GM | Gravimetric (polycarbonate filter, mass sampler 2 lpm Escort ELF): mass (total): 1 m  Real time (portable aerosol spectrometer Grimm 1.109, <1 µm): mass (PM1): 1 m  Real time (SMPS TSI 3936l75, 0.015-0.661 µm): number (<0.1 µm, total): 1 m  Real time (CPC P-track 8525, 0.02-1 µm): number (total): 1m | >2000m^2^ area with general ventilation |
| Hedmer (2014) | 1.3 GSD 2.9  GM | - | Gravimetric (MCE filter, MSA Escort Elf/SKC Aircheck): mass (respiratory): BZ | Mixed area and ventilation  PPE: Mixed |
| Insley (2019) | 0.25 GSD 3.4 (0.9-1.5 m)  0.14 GSD 3.5 (2.1-2.7 m)  GM | 0.039 x10^6^; 2.4 (0.9-1.5 m)  0.040 x10^6^; 2.3 (2.1-2.7 m)  GM | Real time (P-Trak TSI 8525, 0.02-1 µm): number (UFC): 0.9-1.5/2.1-2.7 m  Real time (DustTrak TSI DRX 8533, 0.1-15 µm): mass (total): 0.9-1.5/2.1-2.7 m | 8500/45000m^3^ area with natural ventilated  For concentrations of PM1, PM2.5, PM4, see original article |
| Kirichenko (2019) | - | 0.003-0.0045x 10^6^; NR  (Height 0.8 m/15 min settling)  NR | Real time (Aerotrak particle counter TSI 9306, NR): number (total): 1/3/5 m in length or 0.8/1.8/2.8 m in height from source | 60 m^2^ area with no ventilation  Particles measured after other settling periods or distances: Data not extracted, see original article  For concentrations of different size fractions, see original article |
| Lai (2016) | 0.90 (PM10, environment)  0.049 ±0.032 (PM2.5, personal)  NR | 0.22x10^6^ (SMPS)  0.00089x10^6^ (APS)  NR | Gravimetric (MOUDI, 11 stages 0.056-18 µm): mass (PM10): NR (environmental)  Real time (SMPS, TSI 3936, 0.005-0.16 µm): number (total): NR  Real time (APS, TSI 3321, 0.542-19.81 µm): number (total): NR  Real time (Dust check 1.106 Grimm, NR): mass (PM2.5): NR (personal) | Semi-open area  Data from office not extracted, see original article.  PPE:masks  For environmental mass concentrations of PM0.1 and PM2.5, see original article |
| Lehnert (2012) | 1.5 (inhalable, GSP)  2.5 (inhalable, PGP-EA)  1.3 (respirable, PGP-EA)  median | 0.12x10^6^  median | Gravimetric (cellulose nitrate filters, GSP 3.5, NR): mass (inhalable): BZ  Gravimetric (cellulose nitrate filters, PGP-EA, NR): mass (inhalable/respirable): BZ  Real time (SMPS, TSI, 0.14-0.673 µm): number (UFP): BZ | Mixed area and ventilation  PPE: Mixed – PARPS excluded  Mass conc <LOD with PARPS  For concentration of different welding methods, see original article |
| Lin (2015) | 0.53 (manual)  0.66 (automatic)  mean | - | Gravimetric (MCE filter, IOM 225-70A): mass (inhalable): BZ |  |
| Miettinen (2016) | 0.050 (middle of workshop)  NR | 0.088/0.14x10^6^ SD 0.018/0.038 (BZ)  0.068/0.055 x10^6^ SD 0.006/0.017 (middle of workshop)  mean | Gravimetric (PFTE filter, Dekati CI): mass (<1µm): middle of workshop,  Real time (FMPS/SMPS/CPC, TSI 3091/ TSI 3081/ TSI 3007, 0.0056-0.56/0.0096-0.42/NR µm): number (total): BZ/middle of workshop | 18 460 m^3^ area with general ventilation |
| Sajedifar (2018) | 6.6 (23 cm)  3.8 (BZ, 41 cm)  mean | 0.0038x10^6^ (23 cm)  0.0027x10^6^ (BZ, 41 cm)  mean | Real time (dust monitor, GRIMM 1.106, 0.35 - > 6.5 µm): mass/number (total): 23/41 cm | 4x9x3 m area with no ventilation  For concentrations of different size fractions, see original article |
| Yang (2018) | 4.5±0.31  mean | - | Gravimetric (PVC membrane, Marple 298, < 31 µm): mass (total): BZ | Outdoors with windshield |
| Young (2013) | 0.10/0.19 (BZ, PENS)  0.092/0.14 (1.5m, PENS)  0.11/0.12 (3-5 m, PENS)  0.12 (average, PENS)  0.13 (average, SKC)  mean | - | Gravimetric (coated PTFE/Al filter, PENS, cut off 4 µm):  Mass (PM4): BZ/1.5 m/3-5 m  Gravimetric (PTFE filter, SKC respirable dust aluminium cyclon, NR):  Mass (PM4): BZ/1.5 m/3-5 m | 25x50 m area with general ventilation  PPE: activated-carbon facemasks (BZ)  SKC similar to PENS, see original article |
| Zugasti (2012) | 2.0-5.0  range | - | Gravimetric (glass fibre filter, IOM SKC 225-70A/PGP-GSP 3.5/Button SKC 225-360, NR): mass (total): 1 m | 2x2x2 m area with general and local exhaust ventilation  Button and PGP-GSP 3.5 similar, but IOM differs, see original article |


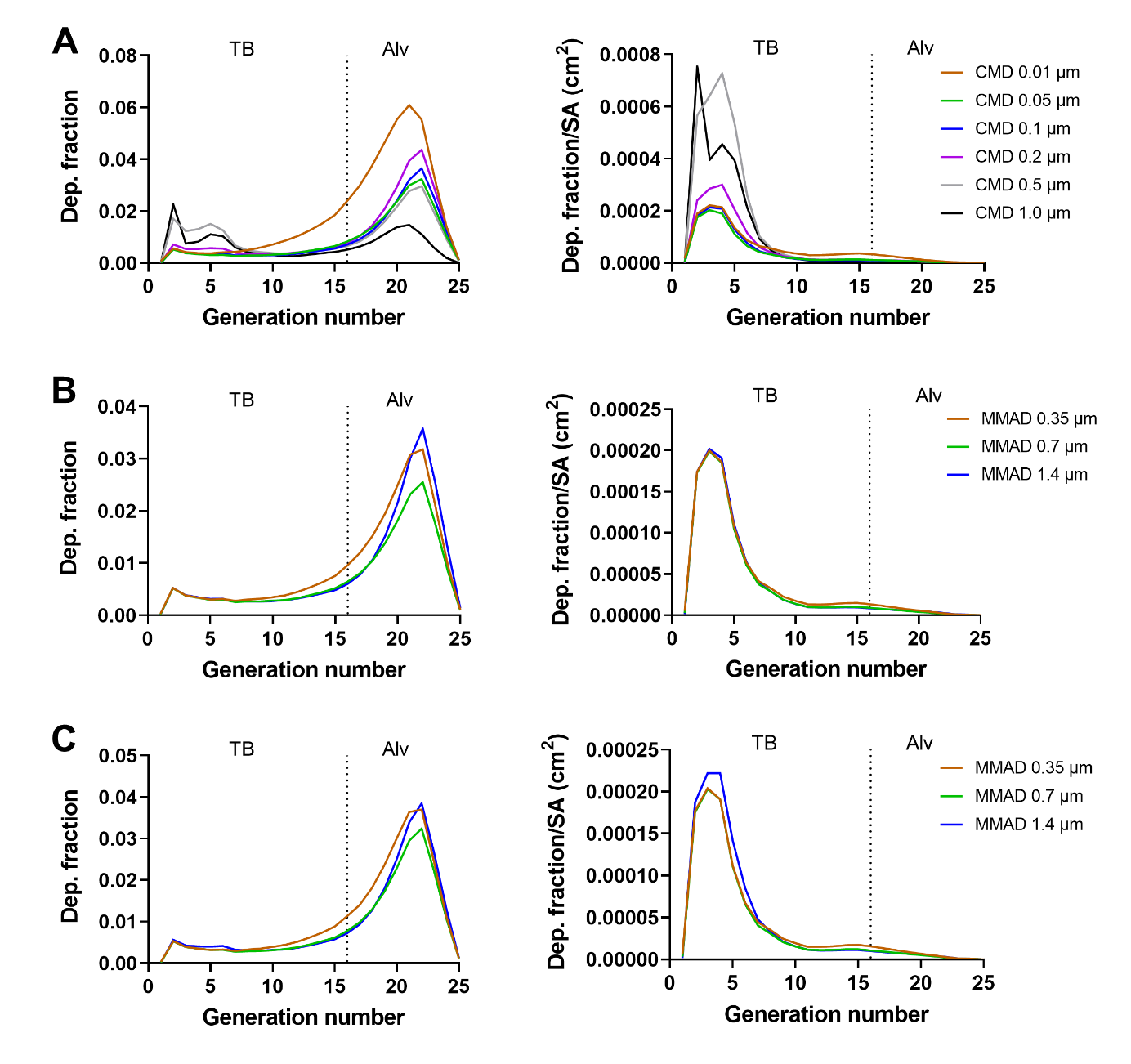


**Figure S1.** Deposition fraction per generation number depending on particle size distribution. Baseline input values were used including the occupational exposure limit concentration of 5 mg/m^3^ and moderate workload. The size distributions were varied from CMD 0.01 to 1 μm (A) or MMAD 0.35-1-4 μm (B,C) with a GSD of 1.2 (B) or 2 (A, C). Results are expressed as deposition fraction over generation number (left) or deposition fraction per surface area over generation number (right).


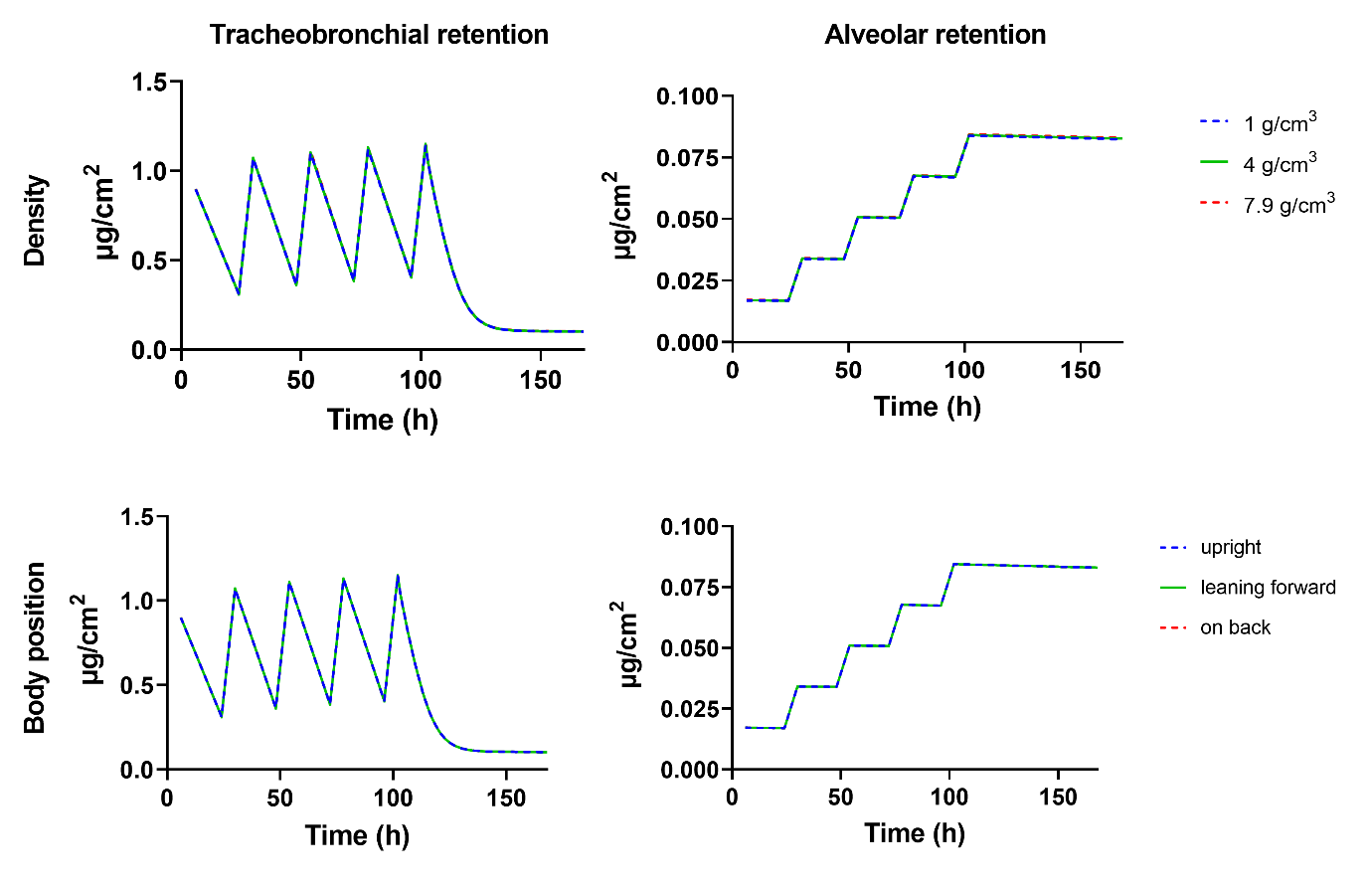


**Figure S2.** MPPD model results tracheobronchial and alveolar retention per surface area versus time (h) during 1 week simulation assuming 6 h of exposure the first 5 days followed by 2 days of only clearance. Baseline input values are marked in green and include a CMD of 0.05 μm (GSD 1.2), the occupational exposure limit concentration of 5 mg/m^3^ and a moderate workload. The influence of a variation of particle density (top) or body position (bottom) were explored.
